# Catastrophic left atrial tear during cryoballoon pulmonary vein isolation following chemotherapy

**DOI:** 10.1016/j.hrcr.2023.04.015

**Published:** 2023-05-05

**Authors:** Gabriel E. Soto

**Affiliations:** SoutheastHEALTH, Cape Girardeau, Missouri

**Keywords:** Atrial fibrillation, Cardiac ablation, Pulmonary vein isolation, Cryoballoon, Mechanical complications, Cardiac perforation, Left atrial tear, Pulmonary vein tear, Cancer, Chemotherapy

**Key Teaching Points**

• Although cryoballoon (CB)-based pulmonary vein isolation (PVI) generally represents a safe and effective treatment modality for atrial fibrillation, it comes with its own set of unique risks.
• Understanding the mechanics of CB operation can help operators understand how inadvertent CB deep-seating within a pulmonary vein (PV) ostium can lead to significant forces being applied to adjacent left atrial/PV tissue, resulting in injury.
• There are limited data regarding the safety and efficacy of PVI in cancer patients.
• Patients who have undergone cardiotoxic and/or immunosuppressive chemotherapies may represent a subgroup of patients at increased risk of mechanical complications for months or even years after completing chemotherapy.

## Introduction

Cryoballoon (CB)-based pulmonary vein isolation (PVI) for the treatment of atrial fibrillation (AF) is a popular alternative to conventional radiofrequency (RF)-based approaches. With more than 1 million CB cases performed worldwide to date, this modality has an outcomes and safety track record similar to RF, with phrenic nerve injury being the leading reported adverse event with CB as compared to RF. Nevertheless, there are a subset of injuries that—while exceedingly rare—have mechanisms unique to CB-based procedures. Herein is described the case of a catastrophic left atrial (LA) tear that occurred at the pulmonary vein ostium during CB ablation, along with an examination of the patient-specific and CB system–specific factors that may have contributed to this complication.

## Case report

The patient was a middle-aged woman in her 50s who initially presented with short episodes of paroxysmal atrial tachycardia and paroxysmal AF. She suffered from severe chronic obstructive pulmonary disease (forced expiratory volume in 1 second 44%, forced expiratory volume in 1 second/forced vital capacity 48%, and diffusing capacity of the lungs for carbon monoxide 47% of predicted values). In the preceding year she had been diagnosed with R-ISS stage II multiple myeloma and underwent a trial of lenalidomide, though this was discontinued owing to side effects. She had a small body habitus with a weight of 55–60 kg and body mass index of 24. Echocardiography demonstrated normal left ventricular systolic function (left ventricular ejection fraction 60%–65%), grade 1 diastolic impairment, and an LA diameter of 2.7 cm. Radionuclide stress imaging demonstrated no evidence of infarct or ischemia.

She was initially placed on a beta-blocker but went on to have increasingly frequent episodes of paroxysmal AF coinciding with initiation of CyBorD (cyclophosphamide/bortezomib/dexamethasone) therapy for her multiple myeloma. She was unable to tolerate flecainide and was placed on low-dose sotalol 60 mg twice daily, which brought her AF under partial control.

Approximately 6 months after undergoing CyBorD therapy, she was placed on pomalidomide, but this was also discontinued owing to side effects. Three months later she underwent an autologous stem cell transplant following induction with melphalan. Her AF burden increased significantly following her transplant and increasing her sotalol to 120 mg twice daily proved ineffective. A shared decision was made to pursue a CB-based PVI, which was scheduled for approximately 5 months after her transplant. Preprocedure computed tomography angiography was not performed.

The patient was brought to the electrophysiology lab, and after induction with general anesthesia vascular access was obtained with placement of decapolar and quadripolar catheters within the coronary sinus and at the His position, respectively. After administration of systemic unfractionated heparin aiming for a target activated clotting time >350 seconds, LA access was obtained with an SL-1 introducer, BRK needle, and SafeSept Transseptal Guidewire (Pressure Products, San Pedro, CA) under fluoroscopic and intracardiac echocardiographic guidance. The SL-1 introducer was exchanged over a 180 cm Extra-Stiff guidewire (Cook Medical, Bloomington, IN) for a FlexCath Advance steerable sheath (Medtronic, Minneapolis, MN).

**Figure 1:**
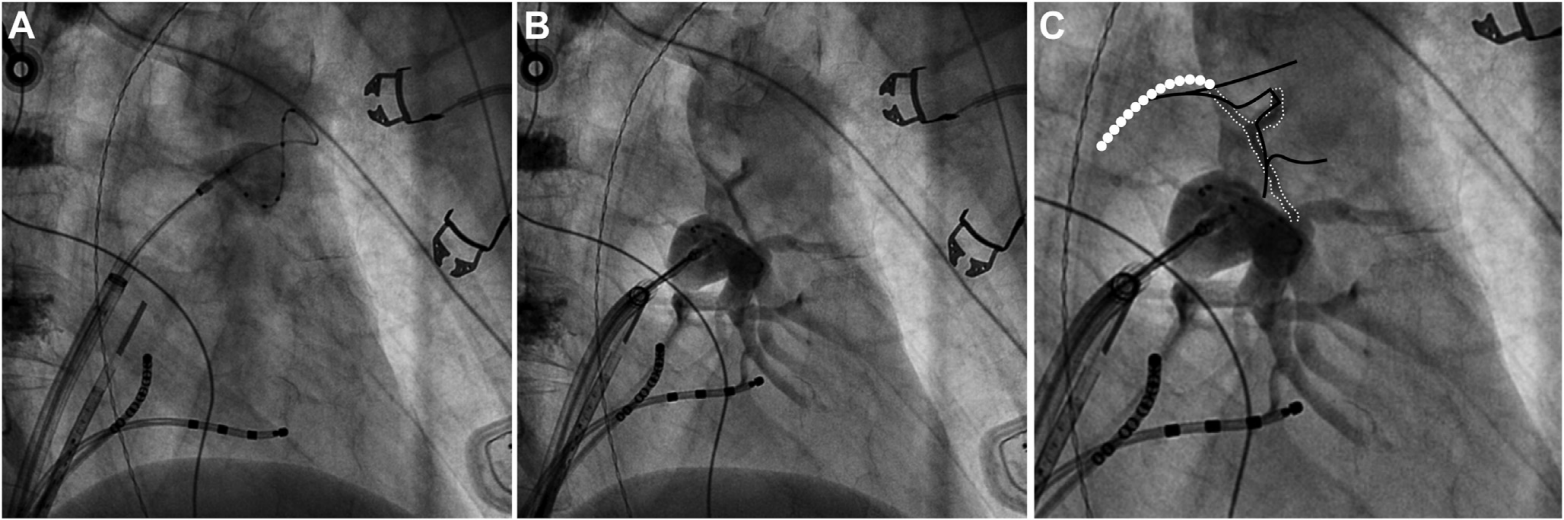
**A:** Right anterior oblique (RAO) (35°) projection demonstrating cryoballoon (CB) position in the left superior pulmonary vein (LSPV). **B:** RAO (35°) projection demonstrating CB position in the left inferior pulmonary vein (LIPV). Note the “Y”-shaped stain above the LIPV and widening of the cardiomediastinal silhouette relative to panel A. **C:** Magnification of image in panel B, with the endocardial staining along the posterior aspect of the LSPV ostium (presumably owing to nontransmural dissection) outlined by small white dots. The approximate location of the transmural tear extending into the left atrium dome is represented by large white dots. The black lines represent the outlines of the LSPV and CB as positioned during LSPV ablation.

An Artic Front Advance Pro 28 mm cryoablation catheter (Medtronic, Minneapolis, MN) was introduced into the LA via the FlexCath sheath, inflated, and advanced to the left superior pulmonary vein (LSPV) ostium over an Achieve Advance mapping catheter (Medtronic, Minneapolis, MN) positioned within the LSPV. After an initial injection of contrast demonstrating occlusion, cryoablation was initiated but stopped after 60 seconds after only reaching a temperature nadir of -34°C. After rewarming to body temperature, the CB was reinflated; however, it was noted to be partially deep-seated within the LSPV, having advanced forward following initial deflation. The CB was gently pulled back toward the LA body until appearing fully expanded under fluoroscopy. Contrast injection demonstrated adequate LSPV occlusion (Figure 1A), and cryoablation was reinitiated. A slight “kick-back” was felt at approximately 30–40 seconds after initiation; no apparent change in catheter position was noted on fluoroscopy, and the LSPV remained opacified with contrast. Cryoablation was continued for a full 180 seconds, reaching a temperature nadir of -53°C.

After balloon deflation, the CB was repositioned to the left inferior pulmonary vein ostium. After contrast injection demonstrated adequate left inferior pulmonary vein occlusion (Figure 1B), cryoablation was initiated. At approximately 30 seconds into cryoablation, anesthesia reported a loss of pulse oximetry and end-tidal CO_2_ waveforms, coinciding with the appearance of a large pericardial effusion on intracardiac echocardiography.

The CB was immediately deflated at 32 seconds and all instrumentation was withdrawn from the left atrium while protamine was administered and cardiopulmonary resuscitation initiated. An emergent pericardiocentesis was performed and approximately 400 cc of hemorrhagic fluid was withdrawn, leading to transient return of spontaneous circulation; however, high output persisted through the drain, indicative of ongoing hemorrhage, and the patient developed pulseless electrical activity while being prepared to go to the operating room. Cardiopulmonary resuscitation resumed as the patient was placed on cardiopulmonary bypass, with surgical exploration ultimately revealing a large 3–4 cm tear extending from the LSPV ostium into the LA dome. The surgeon commented that the “surrounding atrial tissue was noted to have poor overall integrity.” Despite repair of the LA injury, the patient developed a profound coagulopathy and persistent right ventricular failure necessitating placement on extracorporeal membrane oxygenation. Although eventually stabilized from a hemodynamic standpoint, the patient ultimately succumbed to severe anoxic brain injury. Retrospective examination of fluoroscopy images revealed endocardial staining along the posterior aspect of the LSPV that was not appreciated intraoperatively (Figure 1C). It is suspected that the CB was still partially deep-seated within the LSPV after pull-back and upon initiation of the second cryoablation, and that the LA tear occurred when the catheter “kick-back” was felt, but that the CB provided tamponade to the area and masked the injury until CB deflation.

## Discussion

Large tears of the left atrium and pulmonary veins are a rare but potentially catastrophic complication of CB-based PVI.^1^^,^^2^ While deceptively simple to use, the CB system is in fact a remarkably complex device with unique operating characteristics. Notably, the contact forces exerted on atrial tissue surrounding the CB are affected by a number of factors, including user-applied pressure on the CB catheter and introducer sheath as well as CB positioning.

When CB inflation is initiated in the LA body, the internal pressure of the CB (*P*_*CB*_) reaches 3 pounds per square inch (PSI) above atmospheric pressure (ie, *P*_*ATM*_ + 3 PSI, where *P*_*ATM*_ is 14.7 PSI at sea level) within 3 seconds. An automatic safety deflation occurs if *P*_*CB*_ exceeds *P*_*ATM*_ + 5.3 PSI. During this *inflation phase*, *P*_*CB*_ may gradually drift downward owing to the slow atmospheric escape of refrigerant gas from the system.

**Figure 2:**
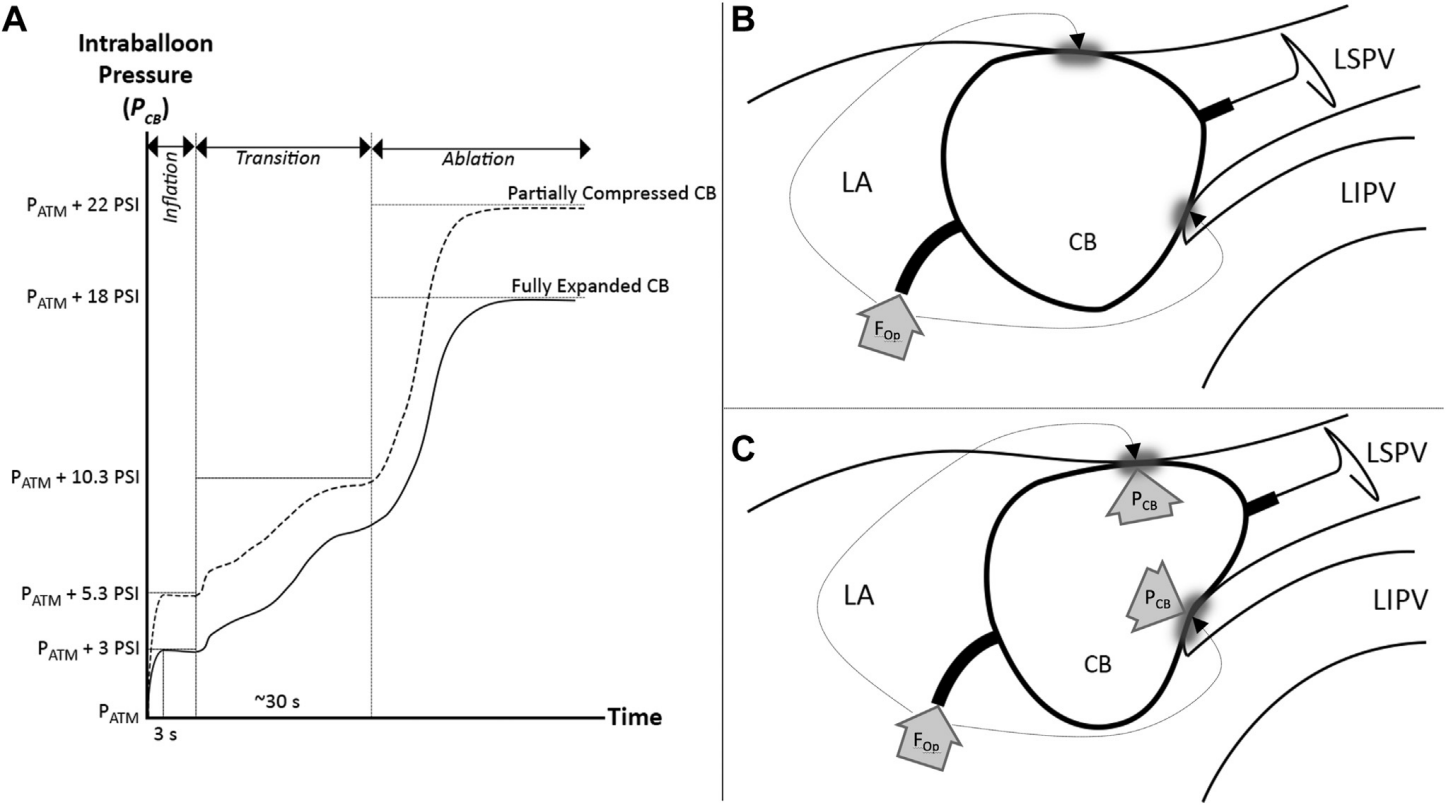
**A:** Internal pressure of the cryoballoon (*P*_*CB*_) as a function of time for a fully expanded cryoballoon (CB) (*solid line*) and a partially compressed CB (*dashed line*) during *inflation*, *transition*, and *ablation* phases. **B:** Optimally positioned CB at the pulmonary vein (PV) ostium. **C:** Deep-seated CB within the PV. For **B** and **C**, the shaded gray zones depict the regions of tissue contact, the arrows labeled *F*_*Op*_ denote the forward force exerted by the operator, and the arrows labeled *P*_*CB*_ depict the forces exerted by the pressurized gas.

Upon positioning of the CB at the pulmonary vein (PV) ostium and initiation of a cryoablation, the CryoConsole enters a *transition phase* that lasts about 30 seconds. During this period, the CryoConsole initiates the injection of liquid refrigerant at 4500 standard cubic centimeters per minute (SCCM), and the return valves begin to open. An automatic safety deflation occurs if *P*_*CB*_ exceeds *P*_*ATM*_ + 10.3 PSI. At the completion of the *transition phase*, the rates of gas injection increase to a target of 7200 SCCM (for the 28 mm Arctic Advance Pro), and the system enters the *ablation phase*, during which *P*_*CB*_ reaches a peak pressure of *P*_*ATM*_ + 18 PSI. The CB pressure characteristics during the *inflation*, *transition*, and *ablation* phases are summarized in Figure 2A. For a fully expanded CB, the force exerted by *P*_*CB*_ is counterbalanced by the CB wall tension. The force transmitted to the LA tissue predominantly results from forward forces exerted by the operator against the CB catheter and sheath (Figure 2B).

With these operational characteristics in mind, what is the impact of having a CB that is deep-seated within a PV ostium during inflation/ablation and compressed by the surrounding LA tissue? The dynamic feedback loops that regulate CB refrigerant gas inflow and outflow significantly attenuate the changes in *P*_*CB*_ that would otherwise result from a reduction in CB volume. Extrinsic compression of the CB to diameters of 26 mm or less results in small increases in peak *P*_*CB*_ during the *inflation* and *transition* phases. Upon moving to the *ablation* phase, *P*_*CB*_ peaks at ∼*P*_*ATM*_ + 20–22 PSI, depending on whether active or passive scavenging is employed on the CryoConsole vacuum line (Figure 2A). In contrast to the case of a fully expanded CB, deep-seating results in the force exerted by *P*_*CB*_ being directly transmitted to the surrounding LA tissue at points of contact. These forces are in addition to any forward forces exerted by the operator (Figure 2C).

Studies in porcine heart models have demonstrated that the minimum force required for LA perforation of healthy (unablated) tissue is 159 g using a 3.5-mm-tip RF catheter.^3^ Using an average contact area of 15.9 mm^2^—derived from measurements using a flat-tipped 3.5 mm RF catheter applied at varying contact angles^4^—this corresponds to a pressure of about 1000 g/cm^2^. Although there are no published data on the risk of LA/PV perforation as a function of force applied over a broad surface area as occurs with CB/LA contact, the upper limit of 22 PSI for *P*_*CB*_ in the case of extrinsic CB compression equates to ∼1550 g/cm^2^; thus, the magnitude of pressures exerted against adjacent LA/PV tissue in the case of CB deep-seating are comparable to those capable of causing perforations with focal RF catheters.

Given the excellent overall safety and performance record of CB-based ablations, the operating characteristics of the CB system appear to be well suited to the vast majority of patients. Even in instances where the CB is inadvertently deep-seated within the PV ostium, the surrounding LA tissue is generally compliant enough to withstand the increased pressure against it, with the primary adverse clinical outcome being an increased risk of collateral cryothermal injury to surrounding tissues. Prior “best practices” have been published offering expert consensus recommendations for minimizing procedural risks, including inflation of the CB within the LA body prior to advancement to the target PV ostium.^5^^,^^6^ Nevertheless, there are patient groups for whom limited outcomes and safety data exist, with cancer patients representing one such population.

Garibaldi and colleagues^7^ recently published a review of PVI outcomes in patients with cancer. Although outcomes and complication rates were comparable to the general population, the 5 reviewed studies collectively included <300 patients and represented only a small subset of cancer types, with breast and genitourinary cancers being among the most represented. Furthermore, there was no information available regarding the timing of PVI procedures and prior cardiotoxic therapies.

The natural history of myocardial injury and repair is well established in the case of ischemic insults, with encapsulation of necrotic tissue and replacement by collagen deposition being largely complete by 4 weeks post infarct.^8^ Most mechanical complications of a transmural myocardial infarction occur between days 3 and 5 post infarct when the tissue is most friable.^9^ In contrast, the progression of myocardial injury and healing in response to cardiotoxic chemotherapeutic agents has been poorly characterized, but may be delayed by weeks or months.^10^ In the case reported herein, the patient was exposed to multiple classes of chemotherapeutic agents (ie, immunomodulators, alkylating agents, and proteasome inhibitors) over a 3-year span ending 5 months prior to her procedure. Most of these agents are known to cause cardiotoxicity through a variety of mechanisms.^11^ The intraoperative findings of highly friable LA tissue suggest that at the time of the procedure, myocardial repair and remodeling was still in its early stages following chemotherapy-induced cellular injury and necrosis, likely contributing to the catastrophic tear that ultimately occurred.

Whether other anatomic features predisposed to this injury is not known, as no preprocedure computed tomography imaging was performed. Nakahara and colleagues^1^ reported a case of a PV laceration involving a CB applied to an elongated PV ostium; however, it is unknown if this generally represents a high-risk structural feature for this complication.

## Conclusion

CB-based PVIs are generally a safe and effective treatment for patients with AF, although they entail some unique inherent risks and a thorough understanding of the operating characteristics of the system is required to minimize complications. Cancer patients represent a population that have not been well represented in clinical trials, with further studies being needed to help determine the optimal timing of PVIs relative to chemotherapy administration.
